# Evaluation of the penetration of intracoronal bleaching agents into the cervical region using different intraorifice barriers

**DOI:** 10.1186/s12903-022-02300-4

**Published:** 2022-06-30

**Authors:** Bugce Sakalli, Fatma Basmaci, Ozlem Dalmizrak

**Affiliations:** 1 Department of Endodontics, Faculty of Dentistry, Near East University, Near East Boulevard, Mersin 10, Turkey; 2 Department of Medical Biochemistry, Faculty of Medicine, Near East University, Mersin 10, Turkey

**Keywords:** Hydrogen peroxide, Intracoronal bleaching, Peroxide leakage, Silicate cement, Monosodium perborate tetrahydrate, Therabase

## Abstract

**Background:**

The present study aimed to make a comparison between the effects of 35% hydrogen peroxide gel (HP) and sodium perborate with distilled water (SP) bleaching agents on the sealing characteristics of glass ionomer cement (GIC), TheraBase, ProRoot MTA and Biodentine intraorifice barriers.

**Methods:**

One hundred and twelve single-rooted mandibular human premolar teeth extracted from young patients (14–25 years) were chosen. Root cement and cementoenamel junction (CEJ) of teeth were examined under a stereomicroscope at 10 × magnification to ensure there was no cement defect or dentin gap in CEJ. After the endodontic access cavities were opened on the occlusal surfaces of the teeth, the working length was determined. Instrumentation of each root canal was performed with a ProTaper Gold rotary system in the determined working length and filled with gutta-percha + AH Plus with a single cone technique using. Root fillings were removed 3 mm short of the CEJ and sealed with one of the following intraorifice barrier materials (n = 30/group): 1. GIC; 2. TheraBase; 3. ProProot-MTA; 4. Biodentine. In each of the sub-groups, either HP or SP was used to perform intracoronal bleaching on days 1, 4, and 7. All outer surfaces of the specimens except the 3 mm cervical region were covered with nail polish and modeling wax layers. Specimens were immersed in a 5 ml Eppendorf tube that contained 2 mL of distilled water. The penetration of peroxide release was measured using the colorimetric ferric thiocyanate method. Statistical analysis of the data was performed with Three-way ANOVA and Tukey’s test (*P* = 0.05).

**Results:**

In the HP groups, GIC showed the greatest peroxide release when compared with other tested groups on day 1 (*P* < 0.05). Biodentine and ProRoot MTA displayed a significantly lower peroxide leakage when compared to GIC and TheraBase on days 1 and 4 (*P* < 0.05). While GIC and TheraBase were used, HP observed higher peroxide penetration when compared with SP on days 1 and 4 (*P* < 0.05).

**Conclusions:**

Peroxide diffusion was significantly influenced by the kind of intracoronal bleaching agents and intraorifice barrier materials used.

## Background

Discoloration of the teeth occurs due to several extrinsic, local and systemic intrinsic factors or as a result of a combination of these factors. Some of the important causes of non-vital tooth discoloration are local intrinsic factors such as trauma, intrapulpal hemorrhage, insufficient removal of the pulp tissue, endodontic materials, post endodontic restorative materials, and root resorption. Especially in anterior teeth, discoloration is strongly associated with esthetic inadequacy. For this purpose, intracoronal bleaching is an effective, safe, and non-invasive procedure among various techniques such as crowns, veneers, or composite resins to improve the esthetic competence of endodontically treated discolored teeth. Intracoronal bleaching using either the thermocatalytic technique, the walking bleach technique or a combination of both [1]. Walking bleach is a commonly preferred intracoronal bleaching method for whitening discolored teeth [2].

Hydrogen peroxide (HP) and sodium perborate (SP) are frequently-used bleaching agents that are used to whiten a discolored tooth. HP is commonly applied directly with a concentration of 30–35% or formed as a result of a chemical reaction of SP. SP is either used with a mixture of distilled water or a combination of HP, which improves the capability of its bleaching effect [3].

Although intracoronal bleaching procedures are conservative, the use of bleaching agents has resulted in complications related to invasive cervical resorption (ICR). Since the mechanism of intracoronal bleaching-induced ICR is not yet fully understood, it is hypothesized that diffusion of bleaching agent into periodontal tissues initiates the inflammatory reaction following the bacterial invasion [2, 4, 5]. HP, due to its low molecular weight and high oxidative power, penetrates easily from the pulp chamber through the dentine tubules to the cervical region. Penetration of HP ions can cause cervical resorption as it provides an acidic pH environment that is optimal for the onset of osteoclastic activity [6]. Furthermore, other complications associated with the use of HP have been reported as increased permeability of dentin, changes in the chemical structure of dentin, and general weakening of the physical properties of dental hard tissue [3, 7]. Thus, to prevent irreversible damage to dental hard and soft tissues in the cervical region, penetration should be limited as possible. This has led to the search for alternative agents that have the same level of effectiveness as conventional approaches but do not have related complications. SP represents a potentially safer alternative for intracoronal bleaching.

The protective barrier material is essential to cover the coronal third of the root canal to prevent or reduce the diffusion of peroxide from bleaching agents into the pulp chamber to the cervical region [1, 8]. Until now, various materials have been recommended for use as a coronal barrier material to prevent peroxide leakage during the intracoronal whitening process, including cavit, amalgam, intermediate restorative material, super-ethoxy benzoic acid (Super-EBA), composite resin, calcium-enriched mixture cement (CEM cement), glass-ionomer cement (GIC), mineral trioxide aggregate (MTA), and Biodentine [9, 10]. GIC has largely been preferred as a coronal barrier material for the coronal leakage of bleaching agents [11].

Recently, calcium silicate-based cements have been employed in endodontic treatment procedures due to their high marginal adaptation, biocompatibility, and ability to allow favorable seal properties [12]. Moreover, these materials are indicated for use in a root-end filling material, pulp capping, apexification, perforation repair, regenerative endodontic procedures, and coronal barrier material [13].

Mineral trioxide aggregate (MTA) is suitable for use as a coronal barrier material to prevent cervical root resorption due to its high marginal adaptability, high concentrations of calcium hydroxide, and resistance to microleakage. However, MTA has some drawbacks such as lack of solubility, long setting time, poor handling, tooth discoloration, and difficulty in removal [14–16].

Biodentine is a compound cement used as a coronal barrier material due to its dentin remineralizing properties, dentin-like mechanical properties, easy use and handling, short setting time, resistance to leakage and non-toxicity. According to the manufacturer, the indications and clinical features are similar to MTA cement with improvements in physical qualities and handling [17–20]. TheraBase is a novel coronal barrier material that is a dual-cure calcium and fluoride-releasing, self-adhesive base-liner. To the best of our knowledge, there are no reported laboratory and clinical studies about the sealing ability of TheraBase during the intracoronal bleaching process [21].

The current research was aimed at evaluating the effects of different frequently-used bleaching agents on the sealing characteristics of various intraorifice barriers. The study tested the two-fold null hypothesis that the type of (1) bleaching agent or (2) intraorifice barrier material does not affect the peroxide penetration.

## Methods

### Tooth selection

G*Power 3.1 software (Heinrich Heine University, Dusseldorf, Germany) was employed to estimate the size of the sample. Accordingly, for the analysis with an alpha-type error of 0.05 and 80% power, it was determined that the size of the sample should be 10 teeth for each group.

The study protocol was approved by the ethics committee of Near East University Health Sciences Ethics Committee (Approval number: YDU-2020/85-1208). The study was conducted in accordance with all the provisions of the World Medical Association Declaration of Helsinki. All methods, involving human participants, were performed in line with the relevant guidelines and regulations of Near East University Health Sciences Ethics Committee. One hundred and twenty single-rooted human mandibular premolars from young patients (14–25 years) were used, the extraction of which had been performed due to periodontal and orthodontic factors. A written informed consent was obtained from all patients and/or their parents in the case of children under 16. Teeth were examined under a microscope at 10 × magnification to ensure there were no cavities, cracks, or restorations. Teeth with more than one canal, anatomical canal variation, resorption, calcification, and apical curvature after buccolingual and mesiodistal examination of specimens on periapical radiographs were not included in this study. Soft and hard tissue residues on the root surfaces were cleaned with ultrasonic tips and stored in a saline solution at room temperature until the experiments were performed.

### Specimen preparation

Traditional endodontic access cavities were opened on the occlusal surfaces of the tooth using 2 mm diameter round and fissure burs (Komet Italia Srl, Milan, Italy) with a high-speed water-cooled. The root canal lengths were measured using a #10 K-file by retracting 1 mm from the root. Instrumentation of the root canals was performed using Protaper Gold rotary files (F3, Dentsply, Maillefer, Ballaigues, Switzerland) with an endodontic motor (X-Smart Plus, Dentsply Maillefer). Irrigation of the root canals was conducted by applying 5.25% sodium hypochlorite (NaOCl) between every file. Final irrigation protocols were as follows: 5.25% NaOCl, 17% ethylenediaminetetraacetic acid (EDTA) (Cerkamed Medical), and saline solution. After drying the root canals by using paper points, gutta-percha was used to fill the root canals (Dentsply, Maillefer, Ballaigues, Switzerland) and AH Plus sealer (Dentsply De Trey, Konstanz, Deutschland). Access cavities were covered with a temporary filling (Dent-a-Cav, Barmstedt, Germany). Each apical end of the tooth was sealed with composite resin. An incubator was used to store the specimens at 35 °C and 100% humidity for 24 h. Following obturation procedures, root fillings were removed 3 mm short of the cementoenamel junction (CEJ) by using a low-speed post drill (LuxaPost; DMG, Hamburg, Germany) with a diameter of 1.5 mm to create space for intraorifice barrier materials.

Division of the specimen into twelve main groups was performed on a random basis (n = 10). The teeth were separated into two different groups (n = 40) with respect to the bleaching agents: HP (35% Hydrogen peroxide gel, Opalescence Endo, Ultradent, South Jordan, UT, USA) and SP (Sodium perborate tetrahydrate powder, Merck, KGaA). The bleaching agent groups were separated into four subgroups with respect to the intraorifice barrier material (n = 10): GIC (Ionoseal, Voco GmbH, Germany); TheraBase (Bisco IncSchaumburg, IL, USA); ProRoot-MTA (Dentsply Tulsa Dental, Tulsa, OK, USA) and Biodentine (Septodont, France). The remaining specimens were divided into four control subgroups (intraorifice barrier materials + distilled water) (n = 10).

The canal orifice of each specimen was filled with the intraorifice barrier materials with a 3 mm thick layer in line with the guidelines of the manufacturer. After the intraorifice barrier materials had been placed, all specimens were kept in an incubator at a temperature of 35 ºC and humidity 100% for three days to permit the complete setting of the materials.

### Bleaching process

**Fig. 1:**
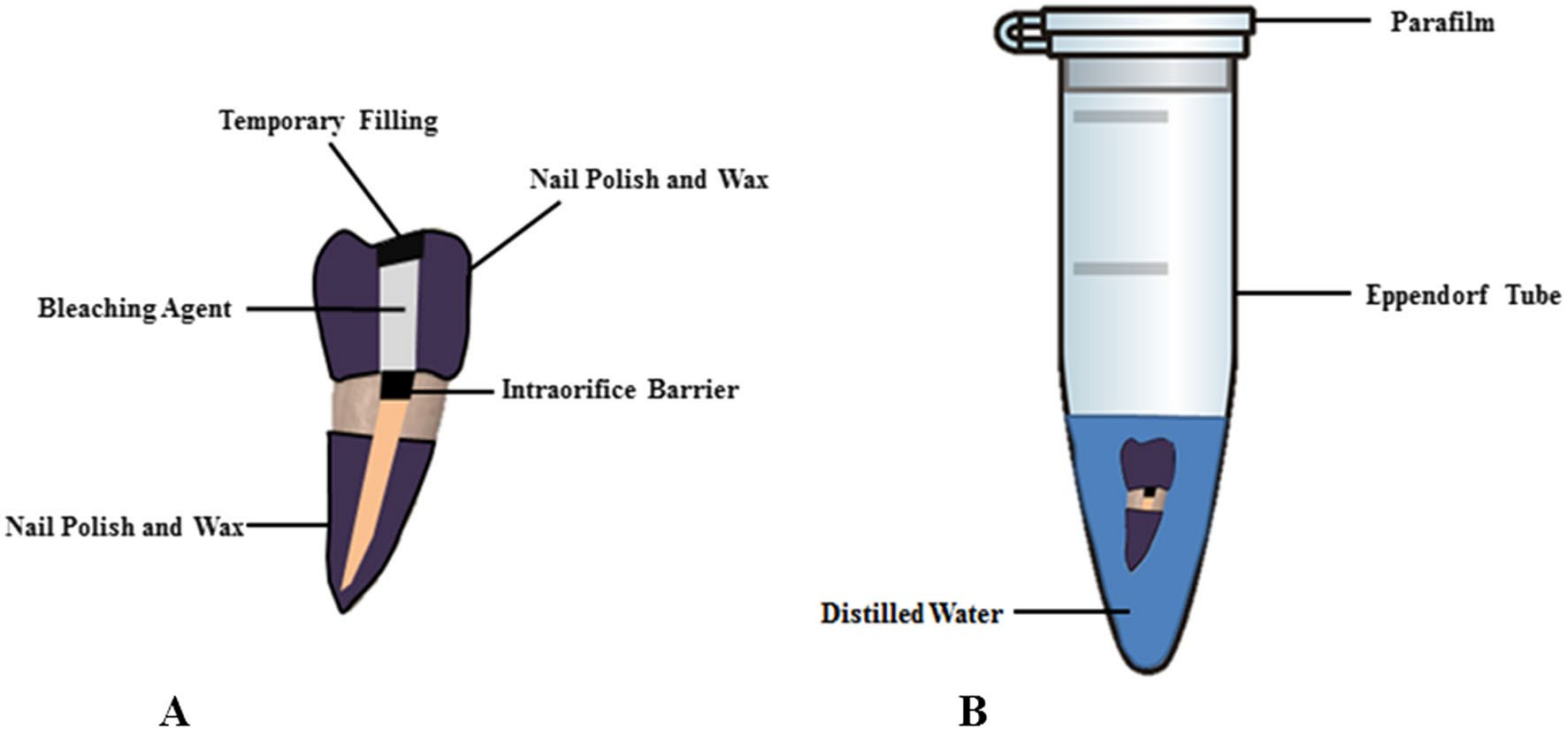
Schematic representation of the tooth (**A**) and the experimental model (**B**)

HP is a ready-to-use bleaching agent in a syringe containing 1.2 ml gel and needle tip. According to the manufacturer's instructions, it approximately 0.03 ml HP was applied to the pulp chamber at with the help of special needle tips. SP is consisted of powder and liquid form. It was mixed in a ratio of 2 g of powder to 1 ml distilled water on the glass slab with spatula to obtain a consistency of wet sand ratio. The mixture was then placed in to the pulp chamber with the aid of an amalgam carrier. After their application to the pulp chamber, top of the bleaching agents were covered with Teflon tape and temporary filling material (Dent-a-Cav, Barmstedt, Germany) was placed over the access cavity. The bleaching materials in all groups were not renewed throughout the experiment at 7 days. In the control group, cotton pellets were wetted with distilled water and placed into the pulp chamber. All outer surfaces of the specimens except the 3 mm cervical region were covered with nail polish and modeling wax layers (BMS Dental, Italy). Specimens were immersed in a 5 ml Eppendorf tube that contained 2 mL of distilled water by micropipette and then kept in an incubator at 35 ºC and 100% humidity (Fig. 1).

### Peroxide penetration analysis

**Fig. 2:**
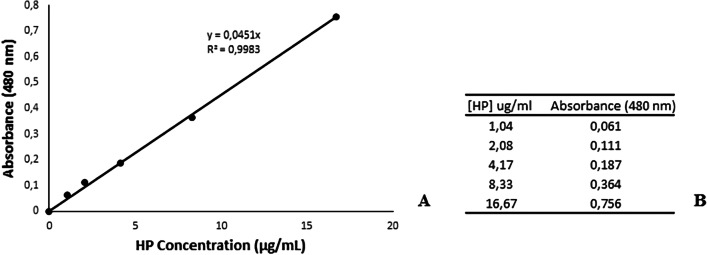
A representative spectrophotometric calibration curve for HP concentration (**A**) and data points for the calibration curve (**B**)

Peroxide penetration was quantified by the colorimetric ferric thiocyanate method [22]. This method relies on the oxidation of ferrous iron (Fe^2+^) to ferric iron (Fe^3+^) in the presence of hydrogen peroxide. Ferric iron then reacts with ammonium thiocyanate to form ferric thiocyanate, a red-orange colored complex that can be measured at 480 nm. The absorbance is directly proportional to the hydrogen peroxide concentration. Briefly, 0.142 gr. of ammonium iron (II) sulfate (AFG Bioscience LLC) was dissolved in 50 µL of distilled water to obtain 10 mM solution. Similarly, 2.5 M ammonium thiocyanate (Riedel–de Haen) was prepared by dissolving 9.515 gr. in 50 µL of distilled water. Standard hydrogen peroxide solutions were prepared by diluting stock (30%) hydrogen peroxide solution (Merck, KGaA) to yield final concentrations of 1.04 µg/mL, 2.08 µg/mL, 4.17 µg/mL, 8.33 µg/mL and 16.67 µg/mL. Two hundred µL of standard or sample was mixed with 54 µL of 10 mM ammonium iron (II) sulfate and 27 µL of 2.5 M ammonium thiocyanate in 96 well plates at room temperature and the absorbances were measured at 480 nm by using a VersaMax Spectrophotometer (Molecular Devices, Microplate Reader, USA). All measurements were made in triplicates and average absorbance values were used in graphs and calculations. A standard calibration curve was plotted by using the absorbances and concentrations of standard solutions. The concentration of hydrogen peroxide within the samples was then ascertained by making a calculation using the equation provided from the calibration curve (Fig. 2). After each analysis, samples were replenished with 2 ml of distilled water.

### Statistical analysis

Data analysis was performed using three-way ANOVA along with Tukey’s multiple comparison test. Computer software was used for the statistical analyses (Jamovi version 1.6; https://www.jamovi.org) (*α* = 0.05).

## Results

Both intracoronal bleaching agents demonstrated peroxide release to the outer surface of the cervical region. The mean ± standard deviation values of the extraradicular peroxide leakage from the experimental and control groups on days 1, 4, and 7 are presented in Tables 1 and 2.

**Table 1:** Mean and standard deviation values of peroxide diffusion (µg/mL) for each experimental group (n = 10). Pairwise comparisons are given with respect to intraorifice barrier (Factor 1) and time frame (Factor 2) for each bleaching agent and control

Groups		Mean ± SD		
Bleaching agent	Barrier	Day 1	Day 4	Day 7
Hydrogen Peroxide (HP)	Biodentine	0.325 ± 0.042^Aa^	0.188 ± 0.069 ^Aa^	0.106 ± 0.049 ^Aa^
	GIC	1.324 ± 0.292^Ab^	0.515 ± 0.226^Bb^	0.103 ± 0.037^Ca^
	ProRoot MTA	0.391 ± 0.218^Aa^	0.224 ± 0.055^ABa^	0.112 ± 0.027^Ba^
	TheraBase	0.819 ± 0.358^Ac^	0.565 ± 0.261^Bb^	0.136 ± 0.023^Ca^
Sodium Perborate (SP)	Biodentine	0.312 ± 0.142^Aa^	0.191 ± 0.056 ^Aa^	0.094 ± 0.015 ^Aa^
	GIC	0.434 ± 0.136 ^Aa^	0.235 ± 0.104 ^ABa^	0.079 ± 0.010^Ba^
	ProRoot MTA	0.277 ± 0.097 ^Aa^	0.187 ± 0.027 ^Aa^	0.073 ± 0.041 ^Aa^
	TheraBase	0.369 ± 0.194 ^Aa^	0.247 ± 0.048 ^ABa^	0.080 ± 0.011^Ba^
Control (Water)	Biodentine	0.034 ± 0.015 ^Aa^	0.026 ± 0.010 ^Aa^	0.018 ± 0.005 ^Aa^
	GIC	0.039 ± 0.013 ^Aa^	0.026 ± 0.007 ^Aa^	0.021 ± 0.005 ^Aa^
	ProRoot MTA	0.048 ± 0.018 ^Aa^	0.030 ± 0.005 ^Aa^	0.026 ± 0.006 ^Aa^
	TheraBase	0.045 ± 0.020 ^Aa^	0.028 ± 0.009 ^Aa^	0.027 ± 0.006 ^Aa^

Means sharing a superscript letter are not significantly different (*p* > 0.05)

Uppercase letters compare means in each row (day factor)

Lowercase letters compare means in each column (intraorifice barrier factor) for each bleaching agent and control

**Table 2:** Pairwise comparisons with respect to bleaching agent (Factor 3) for each intraorifice barrier and each time frame

Groups		Mean ± SD		
Barrier	Bleaching agent	Day 1	Day 4	Day 7
Biodentine	HP	0.325 ± 0.042^a^	0.188 ± 0.069 ^a^	0.106 ± 0.049 ^a^
	SP	0.312 ± 0.142^a^	0.191 ± 0.056 ^a^	0.094 ± 0.015 ^a^
	WATER	0.034 ± 0.015^b^	0.026 ± 0.010 ^a^	0.018 ± 0.005 ^a^
GIC	HP	1.324 ± 0.292 ^a^	0.515 ± 0.226 ^a^	0.103 ± 0.037 ^a^
	SP	0.434 ± 0.136 ^b^	0.235 ± 0.104 ^b^	0.079 ± 0.010 ^a^
	WATER	0.039 ± 0.013^c^	0.026 ± 0.007 ^b^	0.021 ± 0.005 ^a^
ProRoot MTA	HP	0.391 ± 0.218 ^a^	0.224 ± 0.055 ^a^	0.112 ± 0.027 ^a^
	SP	0.277 ± 0.097^ab^	0.187 ± 0.027 ^a^	0.073 ± 0.041 ^a^
	WATER	0.048 ± 0.018 ^b^	0.030 ± 0.005 ^a^	0.026 ± 0.006 ^a^
TheraBase	HP	0.819 ± 0.358 ^a^	0.565 ± 0.261 ^a^	0.136 ± 0.023 ^a^
	SP	0.369 ± 0.194 ^b^	0.247 ± 0.048 ^b^	0.080 ± 0.011 ^a^
	WATER	0.045 ± 0.020^c^	0.028 ± 0.009^b^	0.027 ± 0.006 ^a^

Means sharing the same lower script letter are not significantly different (*p* > 0.05)

Lowercase letters compare means in each column (bleaching agent factor) for each intraorifice barrier for each day

In the HP groups, GIC showed the greatest peroxide release when compared with the other examined groups (*P* < 0.05). There were no statistically significant differences among Biodentine and ProRoot MTA (*P* > 0.05); however, these intraorifice barriers displayed a significantly lower peroxide leakage when compared to GIC and TheraBase on the 1st and 4th days (*P* < 0.05). On the 1st day, TheraBase had significantly lower peroxide diffusion than GIC (*P* < 0.05).

In both SP and control groups, significant differences were not detected between all intraorifice barriers on days 1, 4, and 7 (*P* > 0.05).

A comparison of the mean peroxide release values within both experimental groups demonstrated that the kind of intraorifice barrier used had a significant effect on the release of peroxide (*P* < 0.05). Accordingly, when Biodentine and ProRoot MTA were used, HP and SP bleaching agents showed statistically similar peroxide leakage on days 1, 4, and 7 (*P* > 0.05). However, while GIC and TheraBase were used, HP observed higher peroxide penetration when compared with SP on the 1st and 4th days (*P* < 0.05).

On day 7, there were no significant differences in terms of the leakage of peroxide between the different groups (*P* > 0.05).

## Discussion

In the literature, different periods of peroxide penetration have been reported [22–24]. The current study examined peroxide penetration levels on days 1, 4, and 7. It has been recommended that to achieve successful aesthetic results in the walking bleach method, bleaching agents should be necessarily renewed at intervals of 3–7 days. According to Fick’s second law, although the structure of dentin can allow the diffusion of peroxide, the concentration of peroxide is equal on both dentin sides, and diffusion stops. This expresses the reason for renewing bleaching agents [25].

To obtain the peroxide penetration levels, various methods can be used such as pH changes [26], titration, colorimetric analysis as well as the usage of horseradish peroxidase [27]. In this study, the colorimetric ferric thiocyanate technique was employed due to its simplicity and its frequent use among researchers for analyzing peroxide penetration [22, 28–31].

In the current research, single-rooted mandibular premolars of young patients that had been atraumatically extracted due to orthodontic factors (14–25 years) were used. One of the reasons for preferring these teeth is that they can be found easily in addition to the fact that previous studies have also used similar teeth on peroxide diffusion [30, 32, 33]. Additionally, variations in the relationship and the type of the CEJ are associated with radicular peroxide penetration. Moreover, several researchers have detected that dentine was not exposed to CEJ in premolar teeth, in comparison to others [28, 30].

In the current research, distilled water was used to completely immerse the teeth. However, in earlier studies, this was not performed [28]. As a result of several pilot studies of the present study, the lack of leakage from the coronal and apical roots indicated that it was well covered. Thus, it is clear that the method has an appropriate standardization of peroxide penetration through the CEJ.

The results of this study demonstrated peroxide releases to the outer surface of the cervical region in all experimental groups tested. According to an in vivo study, HP levels below 20 µmol/L (0.68 µg/mL) are considered safe and it is cytotoxic to most living cells when exceeding 50 µmol/L (1.7 µg/mL) [34]. The levels of HP diffused in this study were below this cytotoxic level for all tested groups.

The first null hypothesis was rejected. Our findings revealed that HP showed significantly greater peroxide penetration against the SP in GIC and TheraBase groups on the 1st and 4th days (*P* < 0.05). Previous studies also reported that HP showed the greatest peroxide leakage in contrast to SP when GIC was used as an intraorifice barrier [30, 32, 35, 36]. HP is the main intracoronal bleaching agent, which is a thermally unstable free radical with reduced molecular weight. Thus, it is capable of diffusing through the enamel and dentin to the extraradicular region. Additionally, high amounts of peroxide penetration can be associated with the concentration and liquid form of HP. These could be reasons for the highest diffusion of HP. SP is an intracoronal bleaching agent when it is dry; however, when acid, warm, or air are present, it is broken down into sodium metaborate, hydrogen peroxide, and nascent oxygen. According to these properties, mixing SP with water represents the safest option for use as an intracoronal bleaching agent when compared with HP [1, 24].

Despite previous studies reporting controversial results, it is not yet well understood which intraorifice barrier material is more appropriate to use for endodontically treated teeth. Although several studies have investigated the sealing ability and microleakage of Biodentine and ProRoot MTA, the results are controversial [37–41]. However, no study has compared the amount of peroxide leakage of intracoronal bleaching agents while Biodentine and ProRoot MTA are used as a coronal barrier. We found similar peroxide penetration when Biodentine and ProRoot MTA were used as an intraorifice barrier in the present study (*P* > 0.05).

According to the findings of this study, the greatest peroxide penetration was determined in the HP + GIC group on day 1. The differences between the GIC and TheraBase intraorifice barriers on days 4 and 7 in all groups were not statistically significant. However, these intraorifice barrier materials displayed a significantly higher peroxide diffusion when compared with Biodentine and ProRoot MTA on days 1 and 4 in the HP group. It can be concluded that the intraorifice barrier materials affect the peroxide penetration, leading to rejection of the second null hypothesis. The findings of this study support a previous study, indicating that the leakage of peroxide from GIC was higher than ProRoot MTA on day 1 in both HP and SP groups [33]. The reasons for these results could be related to the formation of the Biodentine and ProRoot MTA which contain hydroxyl apatite at the material-dentine interface. After mixing liquid and powder, these two formulations create small-sized non-structured hydrate gels, which could have a greater ability to reach gaps and diffuse and fit into the dentinal tubules as the dentin surface is wettened [42]. To the best of our knowledge, there is no existing study in the literature about TheraBase.

Despite the promising results regarding the leakage of peroxide from different intracoronal bleaching agents and intraorifice barriers, it should be kept in mind that, due to many inherent drawbacks, in vitro studies cannot precisely simulate in vivo conditions. Therefore, further in vivo studies are necessary to investigate peroxide penetrations of intracoronal bleaching agents from different intraorifice barriers before being recommended for clinical use.

## Conclusion

Based on our results, within the limitations, it was realized that in all experimental groups, although the sealing ability of the intraorifice barrier did not prevent the peroxide leakage entirely, it was reduced. In conclusion, the diffusion of peroxide was significantly affected by the kind of intracoronal bleaching agents and intraorifice barriers used.

## Data Availability

The datasets used and/or analysed during the current study available from the corresponding author on reasonable request.

## Abbreviations

HP: Hydrogen peroxide
SP: Sodium perborate
GIC: Glass ionomer cement
MTA: Mineral trioxide aggregate
ICR: Invasive cervical resorption
CEM: Calcium-enriched mixture
NaOCl: Sodium hypochlorite
EDTA: Etilendiamin tetraasetic acid
CEJ: Cemento enamel junction
Super-EBA: Super‑ethoxy benzoic acid
mM: Millimolar
µL: Microliter
M: Molarity
nm: Nanometer
ml: Mililiter
